# Surveillance of hemorrhagic fever and/or neuroinvasive disease: challenges of diagnosis

**DOI:** 10.11606/s1518-8787.2021055003068

**Published:** 2021-06-17

**Authors:** Leonardo José Tadeu de Araújo, Lorenzo Lang Gonzalez, Lewis Fletcher Buss, Juliana Mariotti Guerra, David Salas Gomez, Camila Santos da Silva Ferreira, Cinthya Santos Cirqueira, Fábio Ghillardi, Steven S. Witkin, Ester Cerdeira Sabino

**Affiliations:** I Instituto Adolfo Lutz Centro de Patologia Núcleo de Patologia Quantitativa São PauloSP Brasil Instituto Adolfo Lutz. Centro de Patologia. Núcleo de Patologia Quantitativa. São Paulo, SP, Brasil; II Instituto Adolfo Lutz Centro de Patologia São PauloSP Brasil Instituto Adolfo Lutz. Centro de Patologia. São Paulo, SP, Brasil; III Universidade de São Paulo Instituto de Medicina Tropical São PauloSP Brasil Universidade de São Paulo. Instituto de Medicina Tropical. São Paulo, SP, Brasil; IV Instituto Adolfo Lutz Centro de Patologia São PauloSP Brasil Instituto Adolfo Lutz. Centro de Patologia. São Paulo, SP, Brasil; V Instituto Adolfo Lutz Centro de Patologia São PauloSP Brasil Instituto Adolfo Lutz. Centro de Patologia. São Paulo, SP, Brasil; VI Weill Cornell Medicine Department of Obstetrics and Gynecology New YorkNY USA Weill Cornell Medicine. Department of Obstetrics and Gynecology. New York, NY, USA

**Keywords:** Autopsy. Hemorrhagic Fevers, Viral, etiology. Arbovirus Infections, mortality

## Abstract

**OBJECTIVE:**

To evaluate the performance of post mortem laboratory analysis in identifying the causes of hemorrhagic fever and/or neuroinvasive disease in deaths by arbovirus infection.

**METHODS:**

Retrospective cross-sectional study based on the differential analysis and final outcome obtained in patients whose samples underwent laboratory testing for arboviruses at the Pathology Center of the Adolfo Lutz Institute, in São Paulo, Brazil.

**RESULTS:**

Of the 1355 adults clinically diagnosed with hemorrhagic fever and/or neuroinvasive disease, the most commonly attributed cause of death and the most common final outcome was dengue fever. Almost half of the samples tested negative on all laboratory tests conducted.

**CONCLUSION:**

The failure to identify the causative agent in a great number of cases highlights a gap in the diagnosis of deaths of unknown etiology. Additional immunohistochemical and molecular assessments need to be added to the post-mortem protocol if all laboratory evaluations performed fail to identify a causative agent. While part of our findings may be due to technical issues related to sample fixation, better information availability when making the initial diagnosis is crucial. Including molecular approaches might lead to a significant advancement in diagnostic accuracy.

## INTRODUCTION

The Brazilian National Health System (SUS) surveillance system is responsible for investigating causes of death related to infectious diseases in the state of São Paulo^1^. When a diagnosis of the cause of death is uncertain, the system performs a post-mortem analysis encompassing all available clinical, laboratory and epidemiological evidence to assess possible etiological agents. This represents the final opportunity to establish the most likely diagnosis and subsequently alert public health officials to initiate improved surveillance measures.

Arboviruses (ARthropod-BOrne virus) are responsible for a large number of different infections with similar clinical manifestations, ranging from mild to severe febrile illnesses, hemorrhagic fever, and neuroinvasive diseases^2^. Several outbreaks have occurred in Brazil due to newly introduced or re-emerging arboviruses^3,4^, each representing a serious public health issue due to difficulties in containment, differential diagnosis and treatment. When caused by non-Arbovirus diseases, laboratory analysis play a pivotal role in the differential diagnosis of hemorrhagic fever (leptospirosis, spotted fever, hantavirus^5^) and neuroinvasive diseases (such as meningitis, herpes, rabies^6^).

This study evaluates the performance of post-mortem laboratory analysis in identifying the causes of deaths associated with hemorrhagic fever and/or neuroinvasive disease of unknown etiology in the state of São Paulo from 2009 to 2019.

## METHODS

### Study Design

This is a retrospective cross-sectional study conducted at the Pathology Center of the Adolfo Lutz Institute between January 2009 and February 2019. Patient records and laboratory results were accessed in the laboratory management systems (GAL and SIGH).

### Referral Pathway

**Figure 1:**
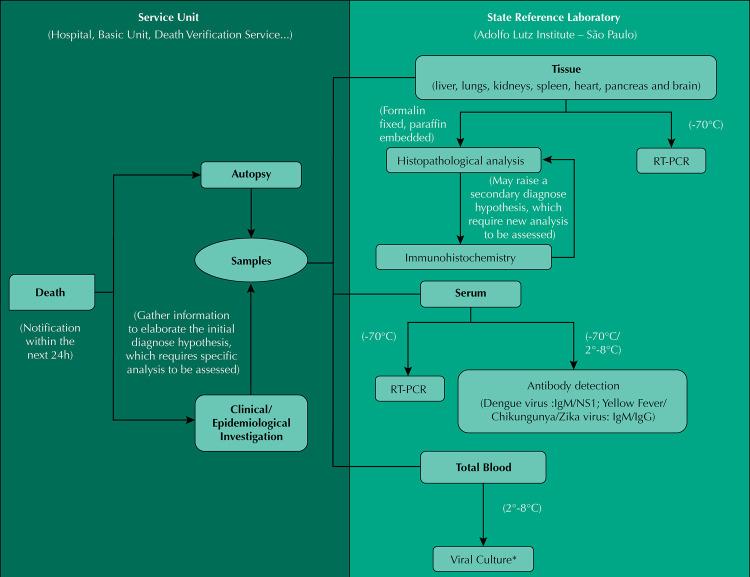
Flow chart for the investigation of post mortem cases related to urban arboviruses at the Adolfo Lutz Institute.

According to recommendations from the Ministry of Health, all suspected deaths must be investigated following the São Paulo State Protocol for the Investigation of Severe Cases and Deaths by Urban Arbovirus^1^, in which samples follow a designated laboratory flow chart (Figure 1). For the purpose of this study, we grouped the cases according to their initial proposed diagnosis, which was based on clinical, laboratory, epidemiological, and necroscopic information – typical basis for post-mortem laboratory analysis. After a multi-organ histopathological analysis, a second possible diagnosis may become evident, which then required further assessment of the immunohistochemistry findings (Figure 1).

### Inclusion Criteria

Our study included patients whose samples underwent laboratory testing for any arbovirus, based on suspected cause of death. Samples were referred for laboratory confirmation if they were related to:

- an urban arbovirus infection outbreak by clinical (regardless of disease progression or the duration of its acute phase) and epidemiological evidence^7^.
- presumed non-arbovirus pathogens, not confirmed by laboratory testing, in which multi-organ histopathological analysis showed findings suggestive of arbovirus infection.

### Clinical Samples and Laboratory Assays

For real-time PCR (qPCR), blood and multi-organ samples (Table 1) were collected during the necroscopic examination, immediately frozen, and sent to the Adolfo Lutz Institute. Multi-organ tissue fragments (Table 1) were also formalin-fixed, paraffin-embedded (FFPE)^1^, and subjected to histopathological assessment and complementary immunohistochemistry. Assays for chikungunya (Evandro Chagas Institute, Para, Brazil), influenza A/H1N1 virus (FluA/H1N1v) (CDC, Atlanta, USA) and *Neisseria meningitidis* (Adolfo Lutz Institute; São Paulo, Brazil) were performed following the standard operating procedures from the Laboratory of Anatomical Pathology of the Adolfo Lutz Institute. Table 1 presents a time lapse of the methods used in the study according to the pathogen and sample analyzed. Patients with clinical diagnosis of acute hemorrhagic fever syndrome (AHFS) were tested for dengue, yellow fever, leptospirosis, hantavirus and rickettsiosis (if there was epidemiological evidence), *N. meningitidis* (if meningitis was suspected by the physician). Patients with clinical diagnosis of severe acute respiratory syndrome (SARS) were tested for FluA/H1N1v.

**Table 1:** Laboratory methods applied to the surveillance of deaths with suspected arbovirus infection.

Pathogen	Time-lapse	Method	Tissue
Dengue virus	2009–2016	Immunohistochemistry^a^	Liver
		qPCR^8^	Liver, spleen, lungs and blood
	2017–2019	qPCR^8^	
Yellow fever virus	2009–2018	Immunohistochemistry^a^	Liver
		qPCR^9^	Liver, spleen and blood
Chikungunya virus	2014	qPCR^10^	Blood
	2015–2018	qPCR^10^	
		Immunohistochemistry^b^	Heart, lungs, muscle
Zika vírus	2016–2018	Immunohistochemistry^b^	Brain, placenta, fetal tissue
		qPCR^11^	Brain, placenta, fetal tissue and blood
Influenza A / type H1N1 virus	2010–2018	Immunohistochemistry^b^	Lungs
		qPCR^c^	
Hantavirus	2009–2018	Immunohistochemistry^b^	Lungs, kidney
		qPCR^12^	
Leishmania spp.	2010–2013	Immunohistochemistry^a^	Liver, spleen, skin
	2014–2015	Immunohistochemistry^a^	
		qPCR^13^	
*Leptospira interrogans*	2009–2018	Immunohistochemistry^a^	Liver, kidney
		qPCR^14^	
*Neisseria meningitidis*	2010–2011	qPCR^15^	Brain
	2012–2015	qPCR^15^	
		Immunohistochemistry^a^	Brain, adrenal glands
	2016–2019	Immunohistochemistry^a^	
Brazilian spotted fever	2009–2018	Immunohistochemistry^a^	Liver, skin
	2010–2018		
		qPCR^16^	Liver, spleen, skin

^a^ Polyclonal primary antibodies and polymer conjugated secondary antibodies.

^b^ Monoclonal primary antibodies and polymer conjugated secondary antibodies.

^c^ CDC/Atlanta/EUA.

### Statistical Analysis

Geographic characterization of the post-mortem cases was performed using the Quantum GIS v. 3.8.3 software; frequency and statistical analysis were performed using Excel (mean and standard deviation [SD]).

### Ethics Statement

This study involved the analysis of routinely collected surveillance data, thus dispensing an Informed Consent Form, according to the Brazilian National Committee for Research Ethics. All procedures were approved by the Adolfo Lutz Institute’s Research Ethics Committee (CAAEE 96138818.0.0000.0059).

## RESULTS

From the initial 1405 cases, we excluded those involving stillbirths and children under one, resulting in 1355 cases for analysis. Most cases (847, 62%) involved males, with a median (range) age of 40 (26–55) years.

Table 2 shows the most common diagnostic hypotheses related to the cause of death in these patients. Most samples were tested for dengue (76%), followed by leptospirosis (47%), yellow fever (29%), rickettsia (28%), hantavirus (24%), influenza (16%), chikungunya (4%) and zika virus (2%), with immunohistochemistry being the most frequently employed detection method (Table 3).

**Table 2:** Most common diagnostic hypotheses related to patient deaths associated with hemorrhagic fever and/or neuroinvasive disease of unknown etiology in the state of São Paulo from 2009 to 2019.

Initial diagnosis	Number of cases n (%)^a^
Dengue	1,034 (76)
Leptospirosis	633 (47)
Yellow fever	398 (29)
Rickettsia (Brazilian spotted fever)	382 (28)
Hantavirus	319 (24)
Influenza	212 (16)
Chikungunya	48 (4)
Zika virus	23 (2)

^a^ Percentages from the 1355 cases included in the dataset. Most cases received more than one differential diagnosis, resulting in the percentages totaling more than 100%.

**Table 3:** Number of cases tested for each etiologic agent and the testing methods employed for diagnosing patient deaths associated with hemorrhagic fever and/or neuroinvasive disease of unknown etiology in the state of São Paulo from 2009 to 2019.

Etiological agent	IHC, n (%)	qPCR, n (%)	IHC and/or PCR^a^, n (%)
Dengue	1,034 (76)	494 (36)	1,130 (83)
Leptospira spp.	905 (67)	25 (2)	912 (67)
Yellow fever	517 (38)	303 (22)	562 (41)
Rickettsia	697 (51)	2 (< 1)	698 (51)
Hantavirus	361 (27)	162 (12)	413 (30)
Influenza	113 (8)	61 (5)	152 (11)
Chikungunya	24 (2)	51 (4)	62 (5)
Zika virus	11 (1)	30 (2)	35 (3)

^a^ Some cases underwent both tests. Percentages from the 1355 cases (full dataset). IHC – immunohistochemistry; qPCR – real-time polymerase chain reaction.

**Figure 2:**
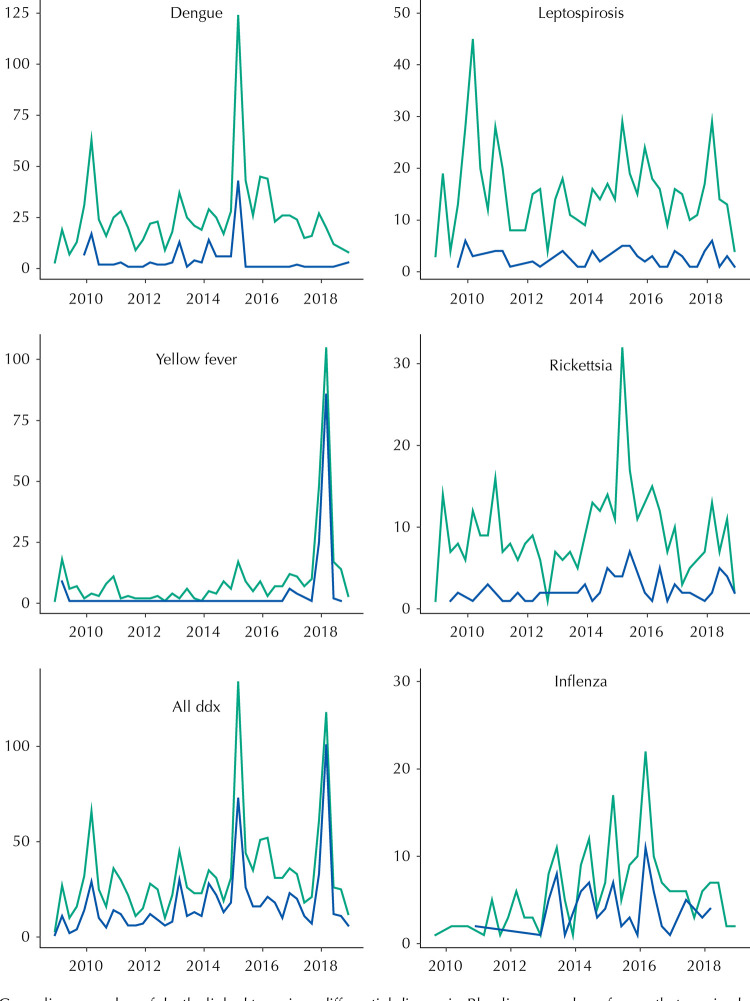
Variation in the number of cases according to the most common initial diagnosis and corresponding final diagnosis of deaths associated with hemorrhagic fever and/or neuroinvasive of unknown etiology in the state of São Paulo from 2009 to 2019.

Table 4 presents the set of differential diagnostic tests employed. Even the high number of tests performed in these sets was insufficient to elucidate most cases, since almost half (48%) tested negative in all laboratory tests conducted, having to rely only on clinical and epidemiological findings for a resolution. Among those with a definitive final diagnosis, 145 (11%) tested positive for dengue, 140 (10%) for yellow fever, 89 (7%) for influenza and 79 (6%) for rickettsia (Table 5). Figure 2 shows the variation between the number of cases according to initial and final diagnosis, depicting the increase in the number of suspected and confirmed cases in specific years, as occurred for dengue, yellow fever and rickettsia outbreaks. The annual seasonal pattern of influenza outbreaks is also evident.

**Table 4:** Set of differential diagnostic tests according to the initial diagnosis of patient deaths associated with hemorrhagic fever and/or neuroinvasive disease of unknown etiology in the state of São Paulo from 2009 to 2019.

Initial Diagnosis (n)	Diagnostic tests performed (IHC and/or PCR)							
	Dengue virus	Leptospira spp.	Yellow fever virus	Rickettsia	Hantavirus	Influenza A / type H1N1 virus	Chikungunya virus	Zika virus
Dengue, (1034)	968 (94)	730 (71)	334 (32)	555 (54)	352 (34)	112 (11)	46 (4)	21 (2)
Leptospirosis, (633)	572 (90)	606 (96)	258 (41)	415 (66)	262 (41)	79 (12)	32 (5)	9 (1)
Yellow fever, (398)	250 (63)	249 (63)	374 (94)	228 (57)	154 (39)	37 (9)	37 (9)	16 (4)
Rickettsia, (382)	348 (91)	342 (90)	194 (51)	349 (91)	193 (51)	34 (9)	29 (8)	5 (1)
Hantavirus, (319)	300 (94)	265 (83)	155 (49)	218 (68)	283 (89)	66 (21)	15 (5)	3 (1)
Influenza, (212)	118 (56)	134 (63)	58 (27)	102 (48)	96 (45)	90 (42)	14 (7)	4 (2)
Chikungunya, (48)	38 (79)	33 (69)	33 (69)	32 (67)	20 (46)	8 (17)	26 (54)	10 (21)
Zika virus, (23)	15 (65)	8 (35)	9 (39)	16 (70)	4 (17)	2 (9)	10 (43)	16 (70)

Note: Percentages are out of the total in column 1 – e.g., 94% of cases in which dengue were the initial diagnoses were tested for dengue.

**Table 5:** Final outcomes of patient deaths associated with hemorrhagic fever and/or neuroinvasive disease of unknown etiology in the state of São Paulo from 2009 to 2019.

Final diagnosis	Number of cases n (%)
Dengue	145 (11)
*Leptospira* spp.	78 (6)
Yellow fever	140 (10)
Rickettsia	79 (6)
Hantavirus	13 (1)
Influenza	89 (7)
Chikungunya	0 (0)
Zika virus	3 (< 1)
Other infectious disease^a^	139 (10)
Other non-infectious disease^b^	20 (1)
No final diagnosis	649 (48)

^a^ Sepsis, gram-positive/negative bacterial infection, lung inflammation/infection, viral hepatitis, fungal infection, adenovirus, enterovirus, herpes, rubeola, schistosomiasis and varicella.

^b^ Disseminated intravascular coagulation, neoplasm, hepatopathy, heart attack.

Green line – number of deaths linked to a given differential diagnosis. Blue line – number of cases that received that final diagnosis (e.g., from the top left panel is the number of cases that received a final dengue diagnosis). In the “All ddx” panel (bottom left) the black line refers to all cases of suspected arboviral infection (whole dataset) and the blue line those cases that received a final diagnosis. The temporal aggregation is on a seasonal level.

## DISCUSSION

Our findings based on the analysis of postmortem data from 1355 adults diagnosed with clinical syndromes suggesting arbovirus infection between 2009 and 2019 revealed that almost half of the cases did not reach a final diagnosis of the causative agent. This highlights the existence of a serious gap in the epidemiological surveillance of endemic and emerging infections in Brazil.

The set of diagnostic tests employed as part of the post-mortem protocol may therefore require additional immunohistochemical and/or molecular evaluation if all laboratory results fail to identify a causative agent. As a result of the post-mortem investigation, about 10% of the final diagnoses differed from the initial diagnoses. Diseases such as bacterial/fungal infection, viral hepatitis, adenovirus, enterovirus, herpes, neoplasms, and heart attacks would have remained undiagnosed if it were not for the histopathological post-mortem investigation procedures. Moreover, the set of diagnostic tests performed on fresh tissue is different from that performed on FFPE tissue. Ideally, both tissue specimens would be collected^17^, but sometimes only one of them is available. In our cohort, some of the FFPE samples lacked a corresponding fresh aliquot available and therefore relied only on immunohistochemical and anatomopathological analysis for diagnosis.

We also have a clear logistics limitation depending on the distance from the collection point to the reference center, along with transport conditions. Although the qPCR assay is able to provide information on the presence of infectious agents at the molecular level, being a very high specific analysis^18^, it is performed only on fresh frozen tissue in public surveillance laboratories. Despite its technical limitations^19,20^, FFPE analysis provides valuable support to immunohistochemical^21,22^ and anatomopathological evidence. Cytological architecture is preserved and the samples can be used to perform molecular tests, such as conventional^23^ or quantitative reverse transcriptase PCR^19,20^. It is important to consider, however, that although immunohistochemistry may reveal the presence of viral antigens within the tissue sample, its specificity may be limited due to the cross-reactivity observed in commercially available antibodies^20,24^ This reinforces the need to use molecular methods as a complementary tool for FFPE.

Despite all efforts to identify an etiological agent, almost half of the cases investigated at the Pathology Center during the study did not receive a final laboratory diagnosis, thus relying solely on clinical and epidemiological information for a tentative conclusion^1^. This is consistent with prior studies reporting that conventional laboratory assays failed to detect a causative agent in approximately 40% of gastroenteritis^27^ cases and 60% of encephalitis^28^ cases. Part of our findings could be attributed to technical issues related to sample fixation^20^, but we also highlight the importance of better information availability when making the initial diagnosis, and having these data available to the reference laboratories (via online laboratory management systems). Lack of clinical data may limit implementing additional laboratory approaches. The more complete and accurate the investigation, the better will be the quality of laboratory surveillance, not only reducing the waiting time for a result, but also limiting the number of unnecessary tests and consequently easing the financial burden on Public Health.

As identified, many other common non-infectious diseases/pathologies (e.g., neoplasms, hepatopathies, vasculitis, hematologic diseases) can mimic arbovirus infections and even hemorrhagic fevers, misleading the diagnostic hypothesis. Another point to consider is that even when laboratory reports are negative for all tested pathogens, the undetected causative agent may be a known species that was simply not included in the laboratory’s testing algorithm. Alternatively, it might involve a truly novel pathogen^29^. Surveillance laboratories must be constantly prepared to identify new pathogens, because their rapid detection is necessary to initiate public health measures.

A potential way to improve laboratory surveillance would be to perform PCR on FFPE samples^30,31^, using specific kits or protocols. Pathogen non-specific molecular methods, such as metagenomic approaches, could have even greater efficacy. Metagenomic sequencing can be used to detect any pathogen without the need for sequence-specific amplification. These sequence data can then be used to predict antibiotic resistance phenotypes^32^, detect co-infections^33^, identify infectious disease outbreaks of unknown causes, and diagnose patients with suspected infections but negative results in conventional tests^34^. This could be a powerful new tool for post-mortem analysis for surveillance purposes. As next-generation sequencing technologies continue to improve and its costs reduced, metagenomic approaches may become increasingly common in public health laboratories. Depending on the clinical and epidemiological information available, a syndromic laboratory approach could be useful for diagnosing hemorrhagic fever deaths of unknown etiology. Multiplex molecular diagnostic assays are able to simultaneously detect and identify the most frequent infectious causes of a single clinical syndrome. They are more accurate, faster and convenient than most techniques previously used in the laboratory^35,36^.

Thus, using a syndromic panel applied to hemorrhagic fever deaths of unknown etiology could contribute to improved surveillance, as has been shown in the diagnosis of respiratory, gastrointestinal, and central nervous system infections^35,36^. Monitoring spatial-temporal trends to identify clusters of negative cases can be used to facilitate early detection of silent infectious disease outbreaks^37^ or even overcome logistical issues affecting sample quality and results, which can occur in non-random yet clustered distributions.

Importantly, the SARS-CoV-2 virus, cause of the COVID-19 acute respiratory disease, was detected in Wuhan, China, by monitoring the emergence of severe cases of “pneumonia of unknown etiology”^38,39^. This underlines the importance of surveillance systems for fatal infectious diseases of unknown etiology to help recognize the emergence of a new outbreak.

## CONCLUSION

Our findings highlight the relevance of a post-mortem laboratory investigation for the accurate diagnosis of arbovirus infections, revealing a gap in the surveillance of deaths from hemorrhagic fever and/or neuroinvasive disease caused by arbovirus infection in the state of São Paulo. The availability of more comprehensive patient data to reference laboratories could improve the quality of laboratory testing. In the future, metagenomic and syndromic laboratory approaches might lead to a significant advance in diagnostic accuracy, thus directly contributing to solving these death causes.
